# HCoV- and SARS-CoV-2 Cross-Reactive T Cells in CVID Patients

**DOI:** 10.3389/fimmu.2020.607918

**Published:** 2020-12-23

**Authors:** Sophie Steiner, Franziska Sotzny, Sandra Bauer, Il-Kang Na, Michael Schmueck-Henneresse, Victor M. Corman, Tatjana Schwarz, Christian Drosten, Désirée J. Wendering, Uta Behrends, Hans-Dieter Volk, Carmen Scheibenbogen, Leif G. Hanitsch

**Affiliations:** ^1^ Institute of Medical Immunology, Charité - Universitätsmedizin Berlin, Corporate Member of Freie Universität Berlin, Humboldt-Universität zu Berlin, and Berlin Institute of Health, Berlin, Germany; ^2^ Department of Hematology, Oncology and Tumor Immunology, Charité - Universitätsmedizin Berlin, Corporate Member of Freie Universität Berlin, Humboldt-Universität zu Berlin, and Berlin Institute of Health, Berlin, Germany; ^3^ Experimental and Clinical Research Center (ECRC), Charité – Universitätsmedizin Berlin, Berlin, Germany; ^4^ Berlin Institute of Health (BIH), Berlin, Germany; ^5^ Berlin Institute of Health Center for Regenerative Therapies (BCRT), Charité University Medicine Berlin, Berlin, Germany; ^6^ Berlin Center for Advanced Therapies (BeCAT), Charité – Universitätsmedizin Berlin, Berlin, Germany; ^7^ Institute of Virology, Charité-Universitätsmedizin Berlin, Humboldt-Universität zu Berlin, Berlin Institute of Health, and German Centre for Infection Research (DZIF), Partner Site Charité, Berlin, Germany; ^8^ Department of Pediatrics, Kinderklinik München Schwabing, StKM GmbH und Klinikum Rechts der Isar, Technische Universität München, Munich, Germany

**Keywords:** common variable immunodeficiency disorder (CVID), coronavirus disease 2019 (COVID-19), T cell response, primary immunodeficiency (PID), severe acute respiratory syndrome coronavirus 2 (SARS-CoV-2), human endemic coronavirus 229E (HCoV-229E), human endemic coronavirus OC-43 (HCoV-OC43)

## Abstract

The inability of patients with CVID to mount specific antibody responses to pathogens has raised concerns on the risk and severity of SARS-CoV-2 infection, but there might be a role for protective T cells in these patients. SARS-CoV-2 reactive T cells have been reported for SARS-CoV-2 unexposed healthy individuals. Until now, there is no data on T cell immunity to SARS-CoV-2 infection in CVID. This study aimed to evaluate reactive T cells to human endemic corona viruses (HCoV) and to study pre-existing SARS-CoV-2 reactive T cells in unexposed CVID patients. We evaluated SARS-CoV-2- and HCoV-229E and –OC43 reactive T cells in response to seven peptide pools, including spike and nucleocapsid (NCAP) proteins, in 11 unexposed CVID, 12 unexposed and 11 post COVID-19 healthy controls (HC). We further characterized reactive T cells by IFNγ, TNFα and IL-2 profiles. SARS-CoV-2 spike-reactive CD4+ T cells were detected in 7 of 11 unexposed CVID patients, albeit with fewer multifunctional (IFNγ/TNFα/IL-2) cells than unexposed HC. CVID patients had no SARS-CoV-2 NCAP reactive CD4+ T cells and less reactive CD8+ cells compared to unexposed HC. We observed a correlation between T cell reactivity against spike of SARS-CoV-2 and HCoVs in unexposed, but not post COVID-19 HC, suggesting cross-reactivity. T cell responses in post COVID-19 HC could be distinguished from unexposed HC by higher frequencies of triple-positive NCAP reactive CD4+ T cells. Taken together, SARS-CoV-2 reactive T cells are detectable in unexposed CVID patients albeit with lower recognition frequencies and polyfunctional potential. Frequencies of triple-functional reactive CD4+ cells might provide a marker to distinguish HCoV cross-reactive from SARS-CoV-2 specific T cell responses. Our data provides evidence, that anti-viral T cell immunity is not relevantly impaired in most CVID patients.

## Introduction

Clinical presentations of coronavirus disease 2019 (COVID-19) are highly variable, ranging from asymptomatic to severe acute respiratory syndrome (SARS). A number of clinical factors with a more than 2-fold increased risk for mortality have been identified and include advanced age, pre-existing respiratory, cardio- and cerebrovascular diseases, hypertension, diabetes and malignancy (1). Ethnicity has also been described as a risk factor for COVID-19 with increased infection rates and worse clinical outcome in Black, Asian and Minority Ethnic individuals (2). According to European Society for Immunodeficiencies (ESID) criteria, patients with common variable immunodeficiency disorder (CVID) have a relevant IgG and IgA +/- IgM deficiency together with reduced class switched memory B cells and/or an impaired specific antibody response to pathogens or vaccination. Due to the inability to mount specific antibody responses to pathogens, patients with CVID are likely at increased risk for severe COVID-19, however clinical data is still very limited (3, 4).

Standard treatment for CVID is IgG replacement therapy, which is effective in infection prevention (5). Because of the novelty of COVID-19, IgG preparations do not contain severe acute respiratory syndrome coronavirus 2 (SARS-CoV-2) IgG yet.

Data on the clinical course of COVID-19 in CVID patients are still very limited. Recently, a fatal outcome was reported and a first report described a moderate to severe course of COVID-19 in 5 CVID patients (4). Authors discussed that CVID patients may be more prone to severe COVID-19 due to preexisting lung inflammatory diseases present in 10% of CVID patients. Data on T cell responses in CVID patients with COVID-19 are currently missing.

Cross-reactivities of T cells against human endemic coronaviruses (HCoV) to SARS-CoV-2 have been proposed and are currently under extensive investigations (6–10). Population studies estimate that approximately 90% express IgG seropositivity to the worldwide circulating endemic HCoV strains, which usually cause milder “common cold” respiratory infections (11). The emerging evidence of pre-existing SARS-CoV-2 reactive T cells shaping the immune response (12), remains to be elucidated in immunodeficient patients. Previous studies provide evidence for normal T cell responses to influenza (vaccine) in CVID patients (13) and to hepatitis B vaccine in X-linked agammaglobulinemia (XLA) patients (14). Here, we aim to characterize the T cell responses to SARS-CoV-2 and two common HCoV strains (229E and OC43) in SARS-CoV-2 unexposed patients with CVID and compare it to T cell responses in unexposed and post-COVID-19 HC.

## Methods

### Human Blood Samples

11 patients with confirmed diagnosis of CVID according to ESID criteria were recruited from the outpatient clinic for immunodeficiencies at the Institute for Medical Immunology at the Charité Universitätsmedizin Berlin (**Table 1A**). Recovered healthy controls (HC) with past COVID-19 had been diagnosed by RT-PCR. The clinical course is described in **Table 1B**. HC without a history of COVID-19 were recruited from laboratory staff and had a negative SARS-CoV-2 antibody test. Blood was drawn from patients and HC in June and July 2020. During the time of our study, the weekly incidence rate of SARS-CoV-2 infections in Berlin was at a level of 0.3–2.0/100 000 inhabitants. The study was approved by the Ethics Committee of Charité Universitätsmedizin Berlin in accordance with the 1964 Declaration of Helsinki and its later amendments (EA2/092/20 from June 4^th^, 2020). All patients and controls gave informed consent.

**Table 1A:** Characteristics of patients and controls.

(A) CVID patients.										
ID	age	sex	IgG [g/l](before RT)	IgA[g/l]	IgM[g/l]	CD4[/nl]	CD8[/nl]	CD19[/nl]	NK[/nl]	EUROClass
**CVID-1**	56	m	0.30	0.06	0.10	0.60	0.90	0.14	0.08	smB-21low
**CVID-2**	60	m	1.66	0.06	0.15	0.32	0.33	0.19	0.10	smB-21norm
**CVID-3**	43	m	3.59	0.06	0.18	0.41	0.36	0.72	0.11	smB-21low
**CVID-4**	29	f	0.00	0.00	2.10	1.02	0.58	0.38	0.11	smB-21low
**CVID-5**	46	f	3.70	0.59	0.40	0.69	0.34	0.24	0.07	smB-21norm
**CVID-6**	56	f	3.12	0.06	0.20	0.45	0.36	0.13	0.14	smB-21low
**CVID-7**	58	m	2.00	0.06	0.05	0.40	0.26	0.12	0.23	smB-21low
**CVID-8**	51	f	3.60	0.25	0.18	0.43	0.23	0.06	0.02	smB-21norm
**CVID-9**	46	m	0.33	0.06	0.05	0.90	0.69	0.16	0.26	smB-21norm
**CVID-10**	74	m	1.30	0.00	0.09	0.50	1.30	0.32	0.26	smB-21low
**CVID-11**	31	m	0.00	0.00	0.00	1.06	0.60	0.00	0.28	B-
**Median**	51		1.66	0.06	0.15	0.5	0.36	0.16	0.11	

CVID, Common Variable Immunodeficiency; NK, natural killer cells; smB, switched memory B cells; norm, normal; B-, patients with equal or less than 1% B cells; f, female; m, male.

**Table 1B:** Characteristics of patients and controls.

(B) post COVID-19 HC.									
ID	Age	Sex	pos. PCR	Time of analysis after first diagnosis by positive PCR [d]	Duration of symptoms [d]	WHO R&D Blueprint ordinal scale	IgG-ELISA [OR ratio]	IgA-ELISA [OR ratio]	PRNT50
**Case-1**	36	w	25.03.2020	51	19	2	1.10	0.42	1:20
**Case-2**	74	m	02.04.2020	60	4	2	1.83	5.03	1:160
**Case-3**	25	w	22.03.2020	70	5	2	1.80	1.66	1:80
**Case-4**	45	w	26.03.2020	75	25	2	7.96	5.02	1:80
**Case-5**	50	w	16.04.2020	48	14	2	2.09	1.56	<1:20
**Case-6**	28	m	28.03.2020	73	n.a.	2	2.48	2.40	1:20
**Case-7**	55	w	09.03.2020	85	13	3	6.23	5.08	1:320
**Case-8**	44	m	26.03.2020	75	8	2	1.31	1.20	<1:20
**Case-9**	22	m	24.03.2020	78	10	2	1.90	3	1:20
**Case-10**	43	m	12.03.2020	95	6	1	2.70	2.43	1:80
**Case-11**	75	m	20.03.2020	19	no symptoms	1	3.43	–	n.a.
**Median**	44			73	10		2.09	2.42	80

PCR, polymerase chain reaction; f, female; m, male; n.a., not applicable; WHO, World Health Organization; PRNT50, the dilution of serum to reduce the number of plaques, of the plaque reduction neutralization test, by 50% compared to the serum free virus.

### Quantification of SARS-CoV-2 IgG

Serum IgG against the N-terminal domain of the spike protein including the immunologically relevant receptor binding domain (RBD) of SARS-CoV-2 was determined by ELISA (EUROIMMUN AG). Neutralizing IgG antibodies were determined by plaque reduction similar as described before (15).

### Flow Cytometric Analysis of Antigen-Reactive T Cells

Peripheral blood mononuclear cells (PBMCs) were isolated from heparin blood samples by density gradient centrifugation, frozen at −80°C and later transferred to liquid nitrogen. Samples from post COVID-19, unexposed HC and CVID patients were simultaneously analyzed. Thawed PBMCs were either incubated with DMSO (background control) or stimulated with 3µg/ml superantigen Staphylococcal enterotoxin B (SEB) (positive control) or 1 µg/ml of peptide pools SARS-CoV-2 Spike Glycoprotein (two vials with N-term and C-Term, PM-WCPV-S-1), SARS-CoV-2 NCAP (PM-WCPV-NCAP-1), HCoV-229E Spike Glycoprotein (two vials with N-term and C-term, PM-229E-S-1) and HCoV-OC43 Spike Glycoprotein S1 (two vials with N-term and C-Term, JPT Peptide Technologies GmbH, Berlin), respectively, for 16h at 37°C and 5% CO_2_. After 2h of stimulation, brefeldin A (BFA) was added as secretion inhibitor. Cells were then stained extracellularly with LIVE/DEAD Fixable Blue Dead Cell Stain Kit (Thermo Fisher Scientific) and lyzed and permeabilized using FoxP3 transcription factor staining buffer set (eBioscience). Afterwards, intracellular staining was performed for CD3 BV650, CD4 PerCp-Cy5.5, CD8 BV510, CD137 PE, CD154 BV421, IL-2 APC, IFNγ BV605, and TNFα AF700 (Biolegend). The stained cells were measured at a CytoflexLX (Beckman Coulter) and analyzed using FlowJo software version 10.6.2 (BD). Reactive CD154+CD137+CD4+ or CD137+CD8+ T cells > 0.005% within total CD4+ or CD8+ T cells and with a ≥ 1.2-fold response of the background control were considered as positive. This threshold corresponds to the range in which 95% of all negative samples are. Unspecific stimulation was excluded by subtracting the background signal of the DMSO sample from the peptide stimulated samples. Single, double (dp) or triple (tp) cytokine producing T cell subsets were analyzed using Boolean combination gates.

### Statistical Analysis

Statistical data analyses were done using GraphPad Prism 6 software. Nonparametric statistical methods were used. Continuous variables were expressed as median and interquartile range (IQR). Univariate comparisons of T cell responses in two independent groups were done using the Mann-Whitney-U test. Distribution of T cell response between the three cohorts was analyzed using a 2 × 2 contingency table. Significance was tested by χ^2^-square test. Correlation between the T cell responses toward the different peptides was analyzed by Spearman’s rank correlation coefficient and linear regression.

A two-tailed p-value of <0.05 was considered statistically significant. Due to multiple testing p-values are considered descriptive.

## Results

### Patient Characteristics and IgG Responses to SARS-CoV-2

11 CVID patients, 11 post COVID-19, and 12 unexposed HC participated in this study. The characteristics of CVID patients are shown in **Table 1A**. The median age for CVID was 51 years (range 29–74), for unexposed HC 35 years (range 25–65) and for post COVID-19 HC 44 years (range 22–75). All CVID patients and 11/12 unexposed and 10/11 Post COVID-19 HC are Caucasian, two are Asian. All CVID patients were under continuous IgG replacement therapy for a minimum of 2 years (median 7, range 2–30 years). Post COVID-19 HC had previous mild COVID-19 (WHO) and a median of 73 days (range 48–95) after diagnosis before T cell analysis (**Table 1B**). Patients with CVID and unexposed HC had no history of COVID-19. All post COVID-19 HC had specific IgG against SARS-CoV-2, while all unexposed HC and CVID patients were seronegative (**Supplementary Figure 1**). In addition, 10 Post COVID-19 HC had neutralizing IgG against SARS-CoV-2.

Further, CD3+, CD4+ and CD8+ T cell frequencies of the three groups are shown in **Supplementary Figure 2**. CVID patients have higher frequencies of CD3+ and CD8+ T cells compared to unexposed HC and post COVID-19 HC, which is already described for CVID patients (16, 17). Frequencies of CD4+ T cells were comparable between the three groups.

### Groups Analysis of SARS-CoV-2 and HCoV-Reactive T Cells

**Figure 1:**
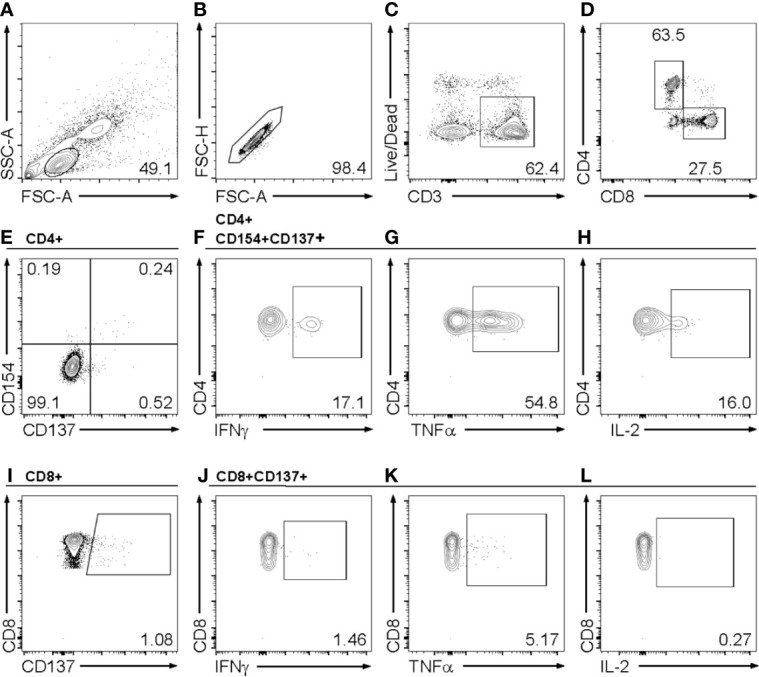
Gating strategy for flow cytometry analysis of activated CD4+ and CD8+ T cells and their cytokine expression profile. Example of a gating strategy in a post coronavirus disease 2019 (COVID-19) healthy control (HC) in response to stimulation with the SARS-CoV-2 C-terminal spike peptide pool. Shown are **(A)** lymphocytes, **(B)** single cells, **(C)** living CD3+ T cells, **(D)** CD4+ and CD8+ T cells, **(E)** activated CD154+CD137+CD4+ T cells, **(F–H)** production of IFNγ **(F)**, TNFα **(G)** and IL-2 **(H)** in CD154+CD137+ activated CD4+ T cells **(I)** and in CD137+CD8+ activated T cells **(H–J)** production of IFNγ **(J)**, TNFα **(K)** and IL-2 **(L)** in activated CD8+ T cells. Single, double (dp) or triple (tp) cytokine producing activated T cell subsets were analyzed using Boolean combination gates.

In order to study the T cell response to SARS-CoV-2 and two common HCoV strains we analyzed the frequency of SARS-CoV-2 spike and NCAP, HCoV-229E and –OC43 spike peptide-reactive CD154+CD137+CD4+ and CD137+CD8+ T cell responses *in vitro* by flow cytometry. Only T cell responses above the threshold of 20% above background activation were included in this study (**Supplementary Table 1**). Cytokine producing capacity of the reactive T cells was assessed by percentages of virus peptide-reactive IFNγ, TNFα and IL-2-producing T cells. **Figure 1** shows the gating strategy in a representative convalescent patient in response to SARS-CoV-2 C-terminal spike peptide pool who had a mild COVID-19 infection.

### CD154+CD137+CD4+ and CD137+CD8+ Activated T Cell Responses to SARS-CoV-2, HCoV-229E and –OC43 and SEB

**Figure 2:**
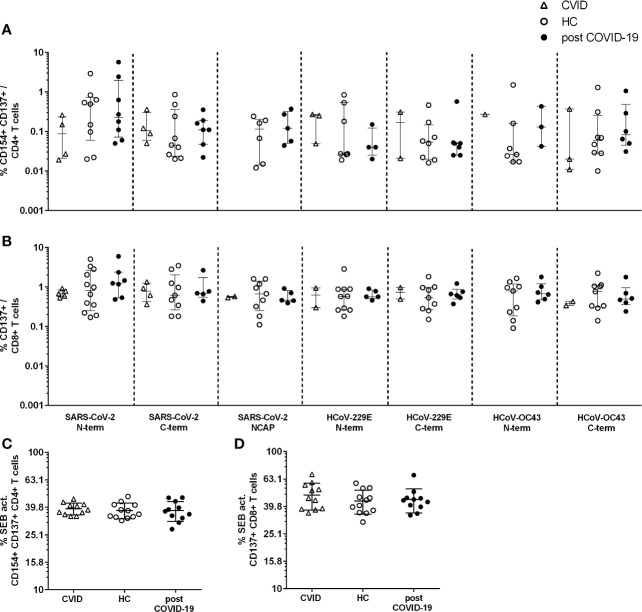
CD154+CD137+CD4+ and CD137+CD8+ T cell response to severe acute respiratory syndrome coronavirus 2 (SARS-CoV-2) and human endemic corona viruses (HCoV) peptides **(A, B)** and Staphylococcal enterotoxin B (SEB). **(C, D)** Peripheral blood mononuclear cells (PBMCs) of CVID (n=11. triangle), HC (n=12. empty black dots) and post-COVID-19 (n=11. filled black dots) were stimulated with 1 µg/ml CoV peptides or 3 µg/ml SEB for 16 h. Frequencies of activated CD154+CD137+CD4+ **(A)** and CD137+CD8+ **(B)** T cells after stimulation with the different CoV peptides. Frequencies of activated CD154+CD137+CD4+ **(C)** CD137+CD8+ **(D)** after stimulation with SEB. Only T cell responses above the threshold of 20% above background activation are shown. CVID patients lacked a response to SARS-CoV-2 NCAP peptide pool in activated CD4+ T cells and are hence not shown **(A)**. Median and interquartile range (IQR) are indicated. Statistical analysis was performed by non-parametric two-tailed Mann–Whitney-U test for comparison of control and patient groups. A p-value ≤ 0.05 was considered as statistically significant.

In 7 of 11 CVID patients, reactive CD4+ T cells against at least one spike peptide pool of SARS-CoV-2 were detectable and in 4 of these 7 also against HCoV-229 and/or –OC43, but none against NCAP (**Figure 2A**). Altogether, there were fewer CD4+ and CD8+ T cells reactive to the 7 spike and NCAP peptide pools in comparison to unexposed HC (p<0.0005 for 1, p<0.005 for 6, p<0.05 for two of 14 peptide responses, **Table 2**). Activated CD4+ T cells reactive against at least one of the spike peptide pools of SARS-CoV-2 were found in 75% of unexposed HC, 81% of post COVID-19 and in 75% and 63% of the HCoVs, respectively (**Figures 2A, B**, **Table 2**, and **Supplementary Tables 1**, **2**). No CVID patient showed a CD4+ T cell response and fewer patients a CD8+ T cell response against SARS-CoV-2 NCAP compared to HC.

**Table 2:** T cell response to peptides of SARS-CoV-2 and HCoV in common variable immunodeficiency disorder (CVID), unexposed and post coronavirus disease 2019 (COVID-19) healthy control (HC).

Peptides	CVID (n=11)	unexposed HC (n=12)	post COVID-19 HC (n=11)
**n of individuals with activated CD4+ T cells**			
**SARS-CoV-2 N-term**	4 (p=0.06)	9	8
**SARS-CoV-2 C-term**	4 (p=0.06)	9	7
**SARS-CoV-2 NCAP**	0 (**)	6	5
**HCoV-229E N-term**	3	7	4
**HCoV-229E C-term**	2 (*)	8	7
**HCoV-OC43 N-term**	1 (*)	7	3
**HCoV-OC43 C-term**	3 (p=0.06)	8	6
**n of individuals with activated CD8+ T cells**			
**SARS-CoV-2 N-term**	5 (**)	12	7
**SARS-CoV-2 C-term**	4 (p=0.06)	9	5
**SARS-CoV-2 NCAP**	2 (**)	9	5
**HCoV-229E N-term**	2 (**)	10	5
**HCoV-229E C-term**	2 (**)	9	6
**HCoV-OC43 N-term**	0 (***)	9	6
**HCoV-OC43 C-term**	1 (**)	9	6

For statistical analysis of CVID vs unexposed HC a 2 × 2 contingency table was used and tested for significance by χ^2^-square test (two-tailed). A two-tailed p-value of p<0.05=* (p<0.005=**; p<0.0005=***) was considered statistically significant. Significant values are bolded.

In CVID patients and HC with positive T cell responses, peptide-reactive CD4+ and CD8+ T cells were found in all three cohorts in a similar frequency (**Figures 2A, B**). Further, activated CD4+ and CD8+ T cells in response to SEB had comparable frequencies in all individuals in the three cohorts (**Figures 2C, D**).

As the median age of the three groups differs, we analyzed if there is an association between age and SARS-CoV-2 spike peptide response. We observed no significant differences in age and response to peptides in all three cohorts (**Supplementary Figure 3**).

Next, we correlated the frequencies of T cells reactive with corresponding peptide pools from SARS-CoV-2 and HCoV. In CVID patients, no correlation analysis could be performed due to too few individuals with reactive T cells. However, all CVID patients with HCoV-reactive T cells had also SARS-CoV-2-reactive T cells. In unexposed HC we found significant correlations for most CD4+ and CD8+ responses against spike peptide pools of N- and C-terminal from all three coronaviruses, suggesting cross-reactive SARS-CoV-2 T cells (**Tables 3A**, **B**). In contrast, in post COVID-19 HC only a correlation of the CD4+ responses against spike of HCoV -OC43, but not with SARS-CoV-2 was found (**Table 3A**). No correlation of frequencies of spike reactive CD4+ T cells with spike specific IgG was found (data not shown).

**Table 3A:** Correlation of the frequency of CD4+ T cells activated by N- or C-terminal spike peptides of SARS-CoV-2 or the endemic corona viruses HCoV-229E and -OC43.

	HCoV-229E N-term	HCoV-229E C-term	HCoV-OC43 N-term	HCoV-OC43 C-term
**healthy controls**				
**SARS-CoV-2 N-term**	**r=0.8649** **p=0.0159** n=7		r=0.7714 p=0.1028 n=6	
**SARS-CoV-2 C-term**		**r=0.9429** **p=0.0167** n=6		**r=0.8214** **p=0.0341** n=7
**HCoV-229E N-term**			*r=0.7827* *p=0.0722* n=6	
**HCoV-229E C-term**				**r=0.8214** **p=0.0341** n=7
**post COVID-19**				
**SARS-CoV-2 N-term**	r=−0.316 p>0.9999 n=4		r=0.5 p>0.9999 n=3	
**SARS-CoV-2 C-term**		r=−0.403 p=0.4333 n=6		r=0.6156 p=0.3 n=5
**HCoV-229E N-term**			n.d. n=2	
**HCoV-229E C-term**				**r=0.8827** **p=0.0444** n=6

Non-parametric spearman correlation was performed. A two-tailed p-value of p<0.05 was considered statistically significant. Significant values are bolded. [r, correlation coefficient; n, number of tested pairs].

**Table 3B:** Correlation of the frequency of CD8+ T cells activated by N- or C-terminal spike peptides of SARS-CoV-2 or the endemic corona viruses HCoV-229E and -OC43.

	HCoV-229E N-term	HCoV-229E C-term	HCoV-OC43 N-term	HCoV-OC43 C-term
**healthy controls**				
**SARS-CoV-2 N-term**	**r=0.8842** **p=0.0013** n=10		**r=0.7167** **p=0.0369** n=9	
**SARS-CoV-2 C-term**		**r=0.9461** **p=0.0013** n=8		**r=0.8929** **p=0.0123** n=7
**HCoV-229E N-term**			**r=0.7699** **p=0.0193** n=9	
**HCoV-229E C-term**				**r=0.7904** **p=0.0251** n=8
**post COVID-19**				
**SARS-CoV-2 N-term**	r=0.6669 p=0.2667 n=5		r=0.7714 p=0.1028 n=6	
**SARS-CoV-2 C-term**		r=0.6 p=0.35 n=5		r=0 p>0.9999 n=4
**HCoV-229E N-term**			r=0.7182 p=0.1667 n=5	
**HCoV-229E C-term**				r=0.1 p=0.95 n=5

Non-parametric spearman correlation was performed. A two-tailed p-value of p<0.05 was considered statistically significant. Significant values are bolded. [r, correlation coefficient; n, number of tested pairs].

### CD4+ and CD8+ Cytokine Responses in Activated T Cells

**Figure 3:**
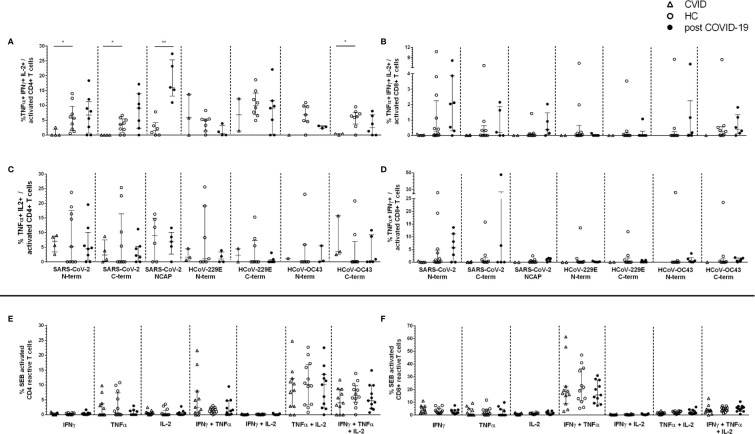
Cytokine expression profiles in activated CD154+CD137+CD4+ and CD137+CD8 T cells. IFNγ, TNFα, or IL-2 single, double (dp) and triple (tp) producing activated CD4+ and CD8+ T cells were analyzed by Boolean combination gating strategy. Cytokine expression profile in triple producing (tp) activated CD4+ **(A)** and CD8+ **(B)** T cells. TNFα+ IL-2+ double producing (dp) CD4+ activated T cells **(C)** and TNFα+ IFNγ+ dp CD8 activated T cells **(D)** in response to CoV peptides (1 µg/ml) are shown. CD4+ **(E)** and CD8+ **(F)** activated T cells in response to Staphylococcal enterotoxin B (SEB) (3µg/ml) were shown. CVID patients lacked a response to severe acute respiratory syndrome coronavirus 2 (SARS-CoV-2) nucleocapsid (NCAP) peptide pool in activated CD4+ T cells and could not be included in the cytokine profile analyses **(A, C)**. Median and interquartile range (IQR) are indicated. Statistical analysis was performed by non-parametric two-tailed Mann–Whitney-U test for comparison of control and patient groups. A p-value ≤ 0.05 was considered as statistically significant. p ≤ 0.05 = *; p ≤ 0.005 = **.

The percentage of cytokine producing T cell responses in CD154+CD137+CD4+ and CD137+CD8+ was analyzed by intracellular staining. Using Boolean combination gating, seven subsets of IFNγ, TNFα and IL-2 single positive, IFNγ/TNFα, IFNγ/IL-2 and IL-2/TNFα double positive (dp) and IFNγ/TNFα/IL-2 triple positive (tp) cells were depicted (**Figure 1** for gating strategy). In the entire cohort, the most frequent CD4+ peptide reactive cytokine subsets were IL-2/TNFα dp and tp T cells (**Figures 3A, C**). CVID patients had significantly lower tp T cells against the spike peptides of SARS-CoV-2 and HCoV-OC43 vs unexposed HC (SARS-CoV-2: N-terminal p=0.0020, C-terminal p=0.036; HCoV-OC43 C-terminal p=0.05; **Figure 3A**), while there were no differences among the post COVID-19 and unexposed HC cohorts. Interestingly, post COVID-19 patients had significantly higher frequencies of tp SARS-CoV-2 NCAP-reactive CD4+ T cells clearly distinguishing them from unexposed HC (p=0.0043, **Figure 3A**). In CVID patients, no CD154+CD137+CD4+ T cell response to SARS-CoV-2 NCAP were found (**Figure 2B** and **Table 2**). The other cytokine subsets are shown in Supplements (**Supplementary Figure 4**). Strongest cytokine responses in CD8+ activated T cells were observed in IFNγ/TNFα dp and tp subsets, but no significant differences among the cohorts were found (**Figures 3B, D**).

Of note, comparable frequencies of SEB-reactive CD154+CD137+CD4+ and CD137+CD8+ cytokine producing activated T cell subsets were observed in all three cohorts, implicating that there is not a general impaired T cell cytokine production in CVID patients (**Figures 3E, F**).

## Discussion

In this study, we provide first evidence of endemic HCoV- and SARS-CoV-2-cross-reactive T cells in CVID patients. However, fewer reactive CD4+ and CD8+ T cells to spike peptide pools and fewer multifunctional CD4+ compared to HC and no NCAP-reactive CD4+ cells were detected.

Our finding of normal frequencies of HCoV and SARS-CoV-2-reactive T cells in a subset of CVID patients is in line with previous studies showing that anti-viral T cell immunity is not relevantly impaired in most CVID patients (13, 18, 19). CVID patients had less frequent T cells reactive against spike peptides of the common cold corona viruses HCoV-229E and -OC43. Possible reasons for this could be that IgG replacement therapy may protect from infections with common cold HCoVs or that patients with CVID avoid contacts with acutely infected persons. Normal T cell reactivity in CVID patients was demonstrated by responses to SEB stimulation, arguing against an obvious underlying T cell defect in non-responders, although an impaired T cell response due to CVID-related immune dysfunction cannot be excluded.

There is increasing evidence, that the majority of HC have T cells reactive to human endemic corona viruses. Our data also provides further evidence for frequent pre-existing T cells reactive against SARS-CoV-2 in unexposed healthy individuals. The presence of cross-reactive T cells to peptide pools of SARS-CoV-2 in unexposed healthy individuals was already reported by different groups ranging from 35% to 90% (7–10, 20, 21). These differences likely depend on the sensitivity of different assays used, and the type of peptide pools. We observed a high correlation of T cells reactive against spike N- or C-terminus of the two HCoVs and SARS-CoV-2 in unexposed but not post COVID-19 HC suggesting cross-reactivity of pre-existing T cells. This finding is in accordance with recent studies from Mateus and Nelde (9, 10). While the RBD is poorly conserved, they provide evidence for homology of many MHC epitopes of the spike protein between HCoV and SARS-CoV-2. We found most unexposed and post COVID-19 HC to have SARS-CoV-2 reactive CD4+ and CD8+ T cells in similar frequencies. In contrast to most other studies, T cell analyses in our convalescent HC was performed median 2.5 months after infection. This could explain why in our study the frequency of SARS-CoV-2 reactive T cells did not differ between COVID-19 recovered patients and unexposed HC.

Virus-specific memory T cells have been shown to persist for many years after infection with SARS-CoV-1 (21–23). In line with these observations, we found that SARS-CoV-2-reactive T cells in convalescent patients acquired a multifunctional (triple positive for INFγ, IL-2 and TNFα) phenotype, which is considered as correlate of protective immunity (24). We found much higher frequencies of tp NCAP reactive CD4+ T cells in post COVID-19 compared to unexposed HC, while high tp spike reactive CD4+ T cells were found in both groups. In CVID patients, no NCAP reactive CD4+ T cells could be detected and spike reactive CD4+ T cells showed little to no tp. A possible explanation for the different cytokine profile is that these CVID patients had contact with HCoV longer time ago. This hypothesis would be supported by comparable frequencies of TNFα single and TNFα/IL-2 dp spike reactive T cells in unexposed CVID and HC belonging to less differentiated and longer lasting memory T cells (16). An alternate explanation would be an impaired ability to mount tp T cells. This is, however, less likely, as we found similar frequencies of tp SEB T cells and tp influenza-specific T cells in CVID vs HC after vaccination (13).

Our cross-sectional study is limited with regards to low numbers of donors and a median higher age in CVID than HC. However, we observed no influence of age on T cell responses. Taken into consideration, that approximately 10 % of patients with mild or asymptomatic SARS-CoV-2 infections fail to mount a detectable antibody response, we cannot exclude that one of our HC had an unrecognized infection with SARS-CoV-2 although it is rather unlikely due to the low number of documented infections in our area in spring 2020. Furthermore, it is of critical importance to evaluate SARS-CoV-2 T cell responses in post COVID-19 CVID patients, too. One limitation of our study is that we could not analyze the T cell response in CVID patients post COVID-19, as until now none of our CVID patients at Charité had COVID-19. Unfortunately, given the continuing spread of the pandemic, SARS-CoV-2 infections in our cohort are very likely to occur and might contribute in evaluating the potential role of the here detected pre-existing SARS-CoV-2 T cells in this patient group. It further remains to be clarified, if SARS-CoV-2 reactive T cells after infection or after vaccination are able to protect or ameliorate the infection in the absence of a humoral immune response as it was reported from previous studies of MERS and SARS-CoV-1 (25–28). The biological relevance of a pre-existing immunity to SARS-CoV-2 remains unclear and could be beneficial or even detrimental. In pandemic influenza H1N1, pre-existing T cell immunity was found to be beneficial (29, 30), so it is tempting to speculate that (cross-) reactive SARS-CoV-2 T cells may provide at least partial protection against COVID-19 disease.

Taken together, our data provides evidence for cross-reactive SARS-CoV-2 cells in a subset of CVID patients as well as a rationale for SARS-CoV-2 vaccination and has implications for the monitoring of vaccine-induced T cell responses.

## Data Availability Statement

The raw data supporting the conclusions of this article will be made available by the authors, without undue reservation.

## Ethics Statement

The studies involving human participants were reviewed and approved by Ethics Committee of Charité Universitätsmedizin Berlin in accordance with the 1964 Declaration of Helsinki and its later amendments (EA2/092/20 from June 4th, 2020). The patients/participants provided their written informed consent to participate in this study.

## Author Contributions

CS, I-KN, MS-H, UB, and LH made substantial contributions to conception and design. LH made patient samples available. SS and SB performed acquisition and analysis of data. SS, FS, LH, and CS performed interpretation of data. CD, VC, and TS and performed analysis of neutralizing IgG. DW and MS-H helped with analyzing data. CS and LH wrote the article. H-DV, MS-H, DW, CD, VC, TS, FS, and SS reviewed the manuscript critically for important intellectual content. All authors contributed to the article and approved the submitted version.

## Funding

This research did not receive any specific grant from funding agencies in the public, commercial, or not-for-profit sectors. We acknowledge support from the German Research Foundation (DFG) and the Open Access Publication Fund of Charité – Universitätsmedizin Berlin.

## Conflict of Interest

VC is named together with Euroimmun on a patent application filed recently regarding detection of antibodies against SARS-CoV-2.

The remaining authors declare that the research was conducted in the absence of any commercial or financial relationships that could be construed as a potential conflict of interest.
