# Supplementary material for: HCoV- and SARS-CoV-2 Cross-Reactive T Cells in CVID Patients
Source: Front Immunol. 2020 Dec 23;11:607918. doi: 10.3389/fimmu.2020.607918 (PMC7785785; doi:10.3389/fimmu.2020.607918)
Supplement: Supplementary file 1 [file DataSheet_1.docx]

Supplementary Material

# Supplementary Figures and Tables

Supplementary tables can be found as Excel sheets under the following names:

**Supplementary Table 1**: Raw data of CD154+CD137+CD4+ and CD137+ CD8+ T cell frequencies

**Supplementary Table 2:** Summary of CD154+CD137+CD4+ and CD137+CD8+ T cell frequencies in CVID, post COVID-19 and unexposed HC

## Supplementary Figures





**Supplementary Figure 1: Anti SARS-CoV-2 ELISA (IgG) of CVID patients, unexposed HC and post COVID-19 HC.** Serum IgG against the N-terminal domain of the spike protein including the immunologically relevant receptor binding domain (RBD) of SARS-CoV-2 analyzed by EUROIMMUN Anti-SARS-CoV-2 ELISA (IgG). All CVID patients and unexposed HC are negative for anti-SARS-CoV-2 IgG. Results are evaluated by calculating a ratio of the OD of the control or patient sample over the OD of the calibrator. A ratio of <0.8 is negative, ratio ≥ 0.8 to <1.1 is borderline, ratio ≥1.1 is positive (dotted line). Median and interquartile range (IQR) are indicated. Statistical analysis was performed by non-parametric two-tailed Mann–Whitney-U test for comparison of control and patient groups. A p-value ≤ 0.05 was considered as statistically significant. p ≤ 0.05 = *; p ≤ 0.005 = **; p ≤ 0.0005 = ***; p < 0.0001 = ****.





**Supplementary Figure 2: T cell frequencies of CVID patients unexposed and post COVID-19 HCs.** Frequencies of CD3+. CD4+ and CD8+ T cell frequencies were analyzed by flow cytometry and calculated as percentage of single, living lymphocytes. CVID patients show higher frequencies of CD3+ **(A)** and CD8+ **(C)** T cells compared to unexposed and post COVID-19 HC. No differences in CD4+ T cells was observed **(B)**. Median and interquartile range (IQR) are indicated. Statistical analysis was performed by non-parametric two-tailed Mann–Whitney-U test for comparison of control and patient groups. A p-value ≤ 0.05 was considered as statistically significant. p ≤ 0.05 = *; p ≤ 0.005 = **

*

*

**Supplementary Figure 3: Analysis of age distribution in SARS-CoV-2 peptide responders and non-responders.** The age of responders and non-responders to SARS-CoV-2 spike peptides was analyzed for reactive CD4+ and CD8+ T cells in CVID **(A)**, unexposed HC **(B)** and post COVID-19 HC **(C)**. No differences of age between responders and non-responders was observed. Median and interquartile range (IQR) are indicated. Statistical analysis was performed by non-parametric two-tailed Mann–Whitney-U test. A p-value ≤ 0.05 was considered as statistically significant. p ≤ 0.05 = *; p ≤ 0.005 = **.

**

**

**Supplementary Figure 4a: Cytokine** **expression profiles in activated CD154+ CD137+ CD4 T cells.** IFNγ, TNFα, or IL2 single and double producing (dp) activated CD4 T cells were analyzed by Boolean combination gating strategy. IFNγ **(A)**, TNFα **(B)**, IL2 **(C)** TNFα + IFNγ **(D)**, IFNγ + IL2 **(E)** producing CD4 activated T cells in response to CoV peptides (1 µg/ml, 16h). CVID patients lacked a response to SARS-CoV-2 NCAP peptide pool in activated CD4 T cells and could not be included in the cytokine profile analyses. Median and interquartile range (IQR) are indicated. Statistical analysis was performed by non-parametric two-tailed Mann–Whitney-U test for comparison of control and patient groups. A p-value ≤ 0.05 was considered as statistically significant. p ≤ 0.05 = *; p ≤ 0.005 = **

**

**

**Supplementary Figure 4b: Cytokine** **expression profiles in activated CD137+ CD8 T cells.** IFNγ, TNFα, or IL2 single and double producing (dp) activated D8 T cells were analyzed by Boolean combination gating strategy. IFNγ **(A)**, TNFα **(B)**, IL2 **(C)** IFNγ + IL2 **(D)**, TNFα + IL2 **(E)** producing CD8 activated T cells in response to CoV peptides (1 µg/ml, 16h). Median and interquartile range (IQR) are indicated. Statistical analysis was performed by non-parametric two-tailed Mann–Whitney-U test for comparison of control and patient groups. A p-value ≤ 0.05 was considered as statistically significant. p ≤ 0.05 = *; p ≤ 0.005 = **
